# Suicide in US Preteens Aged 8 to 12 Years, 2001 to 2022

**DOI:** 10.1001/jamanetworkopen.2024.24664

**Published:** 2024-07-30

**Authors:** Donna A. Ruch, Lisa M. Horowitz, Jennifer L. Hughes, Katherine Sarkisian, Joan L. Luby, Cynthia A. Fontanella, Jeffrey A. Bridge

**Affiliations:** 1 Center for Suicide Prevention and Research, The Abigail Wexner Research Institute, Nationwide Children’s Hospital, Columbus, Ohio; 2 Department of Pediatrics, The Ohio State University College of Medicine, Columbus; 3 Intramural Research Program, National Institute of Mental Health, National Institutes of Health, Bethesda, Maryland; 4 Big Lots Behavioral Health Services and Division of Child and Family Psychiatry, Nationwide Children’s Hospital, Columbus, Ohio; 5 Department of Psychiatry and Behavioral Health, The Ohio State University College of Medicine, Columbus; 6 Department of Psychiatry, Washington University School of Medicine in St Louis, St Louis, Missouri

## Abstract

This cross-sectional study investigates characteristics and trends in suicide rates among US preteens using national mortality data from 2001 to 2022.

## Introduction

Youth suicide is a significant public health concern. In 2021, the National Institute of Mental Health convened a research roundtable series to address the rising rates of suicide in preteens, defined as youths aged 8 to 12 years.^1^ Participants emphasized the need for an improved understanding of suicide risk in preteen subpopulations, particularly those who historically experience health disparities or have been underrepresented in suicide research.^1^ Little is known about the epidemiology of preteen suicide, limiting our ability to inform targeted prevention strategies. We investigated characteristics and trends in suicide rates among US preteens using national mortality data from 2001 to 2022.

## Methods

Data for this cross-sectional study were obtained from the Web-based Statistics Query and Reporting System (WISQARS) where suicide was listed as the underlying cause of death for US preteens from January 1, 2001, to December 31, 2022.^2^ The number of suicide deaths were extracted overall and by sex, race and ethnicity (eMethods in Supplement 1), suicide method, metropolitan or nonmetropolitan area, and region. Trends in were assessed using Joinpoint Regression, version 5.0.2. Negative binomial regression models estimated incidence rate ratios (IRRs) and corresponding 95% CIs to compare period trends using Stata/IC, version 16.0. Confidence intervals that did not include 1.00 were considered statistically significant.

This study was not considered human participant research by the Nationwide Children’s Hospital Institutional Review Board and was therefore deemed exempt from the need for approval or informed consent. We followed the STROBE reporting guideline.

## Results

A total of 2241 preteens died by suicide from 2001 to 2022 (714 [31.9%] female and 1527 [68.1%] male; 162 [7.2%] American Indian or Alaska Native, Asian, or Pacific Islander; 549 [24.5%] Black; 422 [18.8%] Hispanic; and 1530 [68.3%] White). Following a downward trend until 2007, suicide rates significantly increased 8.2% annually from 2008 to 2022, corresponding to a significant increase in the overall rates between 2001 to 2007 and 2008 to 2022 (3.34 to 5.71 per 1 million; IRR, 1.71) (Figure and Table). Analyses revealed significant increases among all subgroups, with the greatest increase in girls (IRR, 3.32), American Indian or Alaska Native, and Asian or Pacific Islander preteens (IRR, 1.99), Hispanic preteens (IRR, 2.06), and firearm suicides (IRR, 2.29).

**Figure.**
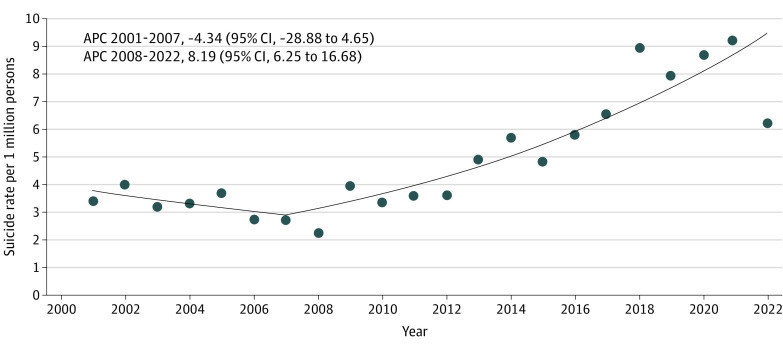
Trends in Suicide Rates for Youth Aged 8-12 Years in the US From 2001-2022 Crude rates per 1 million persons were calculated using Web-based Injury Statistics Query and Reporting System (WISQARS) population estimates. Suicide rate trends were determined using joinpoint regression. Data markers indicate observed rates; suicide rate trends are displayed as solid-colored lines or linear segments connected at the joinpoint or year when the slope of each trend changes significantly. The number and year of joinpoints associated with trends are defined statistically. APC indicates annual percent change for each linear segment trend. A separate joinpoint regression revealed a nonsignificant downward trend from 2021 to 2022 and is not reflected in the figure (APC, 14.40 [95% CI, −24.74 to 3.60]) to highlight the overall significant trend from 2008 to 2022.

**Table.** Period Trends in Suicide Rates for Preteens in the US ^a^

Characteristic	Suicide rates per 1 million persons				Period trend, 2001-2007 to 2008-2022, IRR (95%)^b^
	2001-2007		2008-2022		
	No. of persons	Rate	No. of persons	Rate	
Overall	482	3.34	1759	5.71	1.71 (1.54-1.89)
Sex					
Female	88	1.25	626	4.15	3.32 (2.66-4.15)
Male	394	5.33	1133	7.19	1.35 (1.21-1.51)
Race					
American Indian or Alaska Native, Asian, or Pacific Islander^c^	23	2.60	139	5.17	1.99 (1.28-3.09)
Black	120	4.94	429	8.50	1.72 (1.40-2.11)
White	339	3.05	1191	5.16	1.69 (1.50-1.91)
Ethnicity					
Hispanic	76	2.79	346	5.76	2.06 (1.61-2.65)
Non-Hispanic	406	3.47	1413	5.69	1.64 (1.47-1.83)
Suicide method					
Firearm	90	0.62	440	1.43	2.29 (1.82-2.87)
Hanging or suffocation	373	2.58	1246	4.04	1.56 (1.39-1.76)
Other^d^	19	0.13	73	0.24	NC^e^
Area of residence					
Metropolitan	397	3.24	1445	5.46	1.69 (1.51-1.88)
Nonmetropolitan	85	3.93	314	0.24	1.84 (1.45-2.34)
Region					
Northeast	62	2.41	186	3.74	1.53 (1.15-2.03)
Midwest	123	3.78	434	6.58	1.74 (1.42-2.13)
South	167	3.23	712	6.02	1.87 (1.58-2.22)
West	130	3.78	427	5.75	1.52 (1.25-1.85)

Abbreviations: IRR, incidence rate ratio; NC, not calculated.

^a^ Periods were determined by significant suicide rate trends defined in the Figure.

^b^ Confidence intervals that did not include 1.00 were considered statistically significant.

^c^ Combined into a single group to ensure stable suicide rate estimates.

^d^ Includes poisoning, fall, cut or pierce, drowning, transportation-related, and other means.

^e^ Not calculated because there were too few deaths.

## Discussion

Study findings revealed a significant increase in the suicide rate among US preteens between the 2001-2007 and 2008-2022 periods. Results showing a disproportionate increase in female suicide rates relative to male expand on existing evidence depicting a narrowing of the historically large gap in youth suicide rates between sexes.^3^ Suicide was the 11th leading cause of death in female preteens between 2001 and 2007 and the 5th leading cause of death between 2008 and 2022, while suicide in male preteens ranked consistently as the 5th leading cause of death.^4^

Consistent with previous research,^5^ Black preteens had the highest rates of suicide for both periods, whereas Hispanic preteens had the highest percentage increase. These findings highlight a need to better understand suicide risk among racial and ethnic subgroups, including multiracial individuals who comprise the fastest-growing racial group in the US.^6^ While hanging or suffocation was the predominant method of suicide for the entire period, the largest increase in preteen suicides was by firearm.

This study was limited by potential misclassification of suicides as other causes of death. This misclassification, coupled with a lack of more specific racial and ethnic categorizations, also limits the accuracy of suicide statistics and our knowledge of suicide trends. Additionally, we were unable to examine suicide data through an intersectionality lens, such as racial and ethnic differences by sex, due to small cell counts in WISQARS.^2^

This study provides a foundation for future research to explore unique factors associated with preteen suicide. The findings also support the need for culturally informed and developmentally appropriate prevention efforts that emphasize robust risk screening and lethal means restriction.

## Notes

Supplement 1. **eMethods.** Race and Ethnicity Reporting

Supplement 2.
Data Sharing Statement
