# Supplementary material for: Suicide in US Preteens Aged 8 to 12 Years, 2001 to 2022
Source: JAMA Netw Open. 2024 Jul 30;7(7):e2424664. doi: 10.1001/jamanetworkopen.2024.24664 (PMC11289692; doi:10.1001/jamanetworkopen.2024.24664)
Supplement: Supplement 1. — eMethods. Race and Ethnicity Reporting [file jamanetwopen-e2424664-s001.pdf]

Supplementary Online Content

Ruch DA, Horowitz LM, Hughes JL, et al. Suicide in US preteens aged 8 to 12 years, 2001 to 2022. *JAMA Netw Open*. 2024;7(7):e2424664.
doi:10.1001/jamanetworkopen.2024.24664

eMethods. Race and Ethnicity Reporting

This supplemental material has been provided by the authors to give readers additional information about their work.

© 2024 Ruch DA et al. *JAMA Network Open*.

eMethods. Race and Ethnicity Reporting

Race and ethnicity were categorized based on the Office of Management and Budget (OMB) Standards for the Classification of Federal Data on Race and Ethnicity and summarized into the following reporting groups: American Indian /Alaska Native, Asian/Pacific Islander, Black, Hispanic, and White. Race and ethnicity were assessed to identify potential differences in suicide trends across racial and ethnic groups to better inform targeted intervention strategies.

© 2024 Ruch DA et al. *JAMA Network Open*.
