# Supplementary material for: Suicide in US Preteens Aged 8 to 12 Years, 2001 to 2022
Source: JAMA Netw Open. 2024 Jul 30;7(7):e2424664. doi: 10.1001/jamanetworkopen.2024.24664 (PMC11289692; doi:10.1001/jamanetworkopen.2024.24664)
Supplement: Supplement 2. — Data Sharing Statement [file jamanetwopen-e2424664-s002.pdf]

Data Sharing Statement

Ruch. Suicide in US Preteens Aged 8 to 12 Years, 2001 to 2022. *JAMA Netw Open*. Published July 30, 2024. doi:10.1001/jamanetworkopen.2024.24664

Data
Data available: No

Additional Information
Explanation for why data not available: N/A - publicly available data used in study
